# Adult granulosa cell tumor with rare pulmonary presentation

**DOI:** 10.1016/j.rmcr.2025.102185

**Published:** 2025-02-26

**Authors:** Mark G. Evans, Anthony Crymes, Adam Bedeir, David R. Braxton, Matthew J. Oberley, Robert T. Muldoon

**Affiliations:** a Caris Life Sciences, Phoenix, AZ, USA; b Keck School of Medicine of the University of Southern California, Los Angeles, CA, USA; c Basis Phoenix High School, Phoenix, AZ, USA; d Hoag Family Cancer Institute, Newport Beach, CA, USA; e Genesis Cancer and Blood Institute, Hot Springs, AR, USA

**Keywords:** Adult granulosa cell tumor, *FOXL2* alteration, Pulmonary presentation, Platinum-based chemotherapy, Calretinin, α-inhibin

## Abstract

Adult granulosa cell tumor (GCT) is an uncommon sex cord-stromal neoplasm of the ovary. It typically demonstrates locoregional spread, and disease outside of the pelvis as metastasis is uncommon. Only a rare case of primary thoracic adult GCT has been documented, and here we describe another occurrence with unusual pulmonary presentation, thereby expanding the anatomic distribution across which adult GCT could potentially arise.

## Highlights

• Adult granulosa cell tumor (GCT) can demonstrate unusual primary pulmonary presentation with no evidence of pelvic disease.
• Such rare cases are notable for locoregional recurrence during their treatment course.
• This report of primary thoracic adult GCT expands the anatomic spectrum over which this cancer type is thought to originate.

## 1 Introduction

Adult granulosa cell tumor (GCT) is rare, constituting only 2–5% of neoplasms that arise from the ovary [1]. It originates from granulosa cells and is associated with pathogenic alterations in *FOXL2* [2]. Adult GCT typically follows an indolent disease course with good prognosis. Distant spread is rare and, instead, recurrence typically occurs locally within the pelvis; however, tumor occurring within the liver, bones, and lungs has been reported [[3], [4], [5]]. In particular, disease within the thorax would likely encourage efforts to locate a gynecologic primary site, and metastasis would be the assumed clinical scenario. However, one primary lung GCT has been documented previously [6], and here we describe another patient whose cancer was notable for its pulmonary presentation.

## 2 Case report

**Fig. 1:**
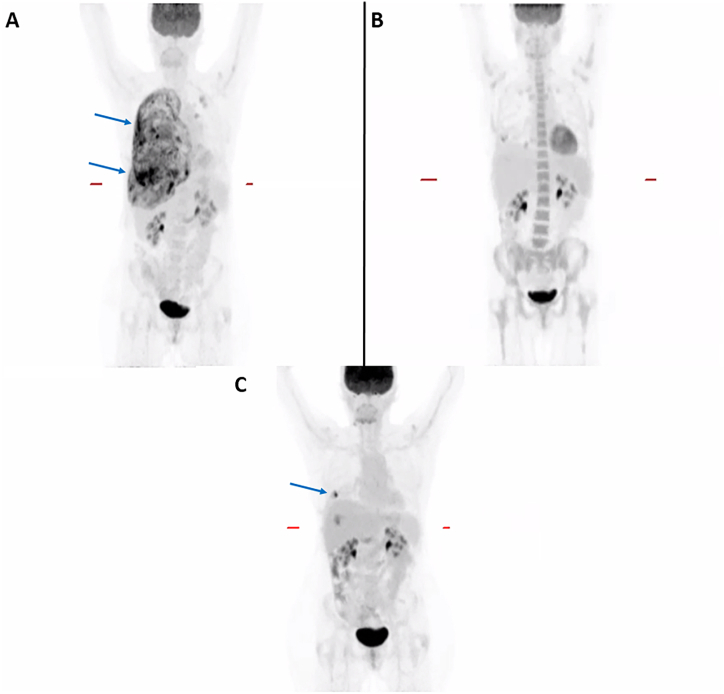
Positron emission tomography–computed tomography (PET/CT) performed at presentation (A) revealed diffuse right-lung inflammation and discrete pleural nodularity (arrow), which demonstrated complete resolution upon treatment with carboplatin, pemetrexed, and pembrolizumab (B). Local pleural recurrence (arrow) was unfortunately confirmed by repeat PET/CT imaging obtained three months post-treatment (C).

A 60-year-old, never-smoker female presented with stabbing pain of the right chest wall and right upper abdomen, in addition to mild shortness of breath, intermittent fevers, and 30-pound weight loss while taking tirzepatide. She underwent hysterectomy and salpingo-oophorectomy approximately 40 years earlier following childbirth and is without history of a prior malignant diagnosis. Computed tomography (CT) revealed a large right-sided pleural effusion and pleural nodularity concerning for lung cancer. Subsequent positron emission tomography–computed tomography (PET/CT) confirmed extensive right pleural disease (Fig. 1A).

**Fig. 2:**
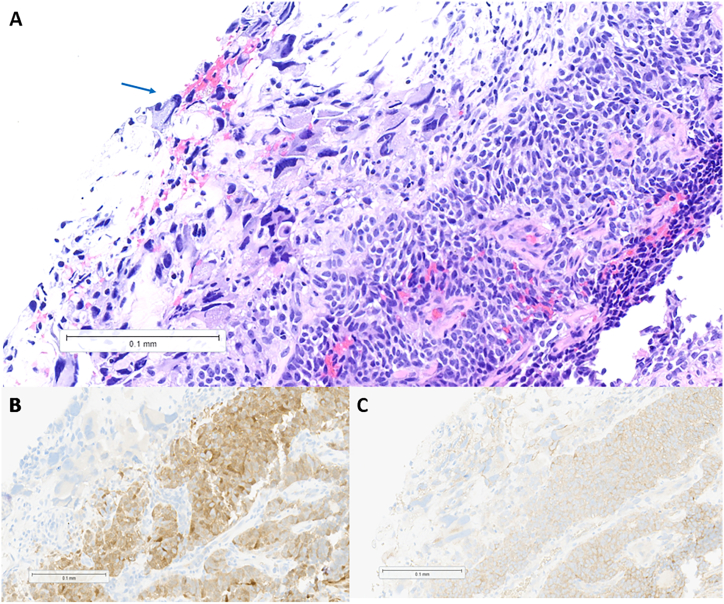
Microscopic examination of the patient's tumor reveals a diffuse proliferation of epithelioid cells with moderately condensed nuclear chromatin, conspicuous nucleoli, and ample eosinophilic cytoplasm (A, hematoxylin and eosin staining, 200x magnification). Focal giant cells with markedly pleomorphic nuclei are noted (arrow). The neoplastic cells demonstrate cytoplasmic staining for calretinin (B, 200x magnification) and for α-inhibin (C, 200x magnification) by immunohistochemistry.

Initial thoracentesis was non-diagnostic, but repeat pleural fluid sampling revealed a malignant epithelial neoplasm consisting of scattered giant cells with marked nuclear pleomorphism (Fig. 2A). By immunohistochemistry, the cells were positive for progesterone receptor, calretinin (Fig. 2B), and α-inhibin (Fig. 2C), with weak expression of AE1/AE3 and CD99; PD-L1 (clone 22c3) testing revealed a combined positive score of 20. The tumor specimen underwent whole exome and whole transcriptome sequencing (Caris Life Sciences, Phoenix, AZ), which revealed pathogenic or likely pathogenic alterations in *TP53* (p.N239S), *ERCC2* (p.Y72C), *TERT* (c.-146C > T), *MUTYH* (p.Y179C), and *FOXL2* (p.C134W). MI GPSai, a Genomic Prevalence Score developed by Caris Life Sciences that uses machine learning with genomic and transcriptomic data to elucidate tumor origin [7], predicted a diagnosis of granulosa cell tumor (GCT). Overall, the constellation of findings was most consistent with a primary pulmonary adult GCT. Initially, the patient received a combination of carboplatin, paclitaxel, and pembrolizumab, with paclitaxel discontinued after two cycles due to an adverse reaction. Once the final diagnosis of GCT was confirmed, treatment was transitioned to two cycles of carboplatin, pemetrexed, and pembrolizumab followed by maintenance pembrolizumab; repeat PET/CT imaging was consistent with a complete response (Fig. 2B).

## 3 Discussion

Our case represents a rare occurrence of adult GCT arising within the thoracic cavity. Given the unusual presentation, important diagnostic considerations were made. Immunohistochemistry confirmed expression of calretinin and α-inhibin, which have a reported specificity of 85 % and 99 %, respectively, for identifying sex cord-stromal tumors [8]. In addition to the compelling evidence provided by gene expression prevalence scoring, the presence of a pathogenic p.C134W *FOXL2* alteration was consistent with adult GCT. Pathogenic changes in this gene are thought to disrupt normal granulosa cell differentiation and alter usual hormone production from ovarian follicles, with approximately 97 % of adult GCTs harboring *FOXL2* alterations [2]. The origin of this uncommon tumor within the thorax of our patient is unclear, but one might speculate that ectopic Müllerian tissue, which potentially implanted during development, could have served as a possible primary site.

Surgical excision is typically pursued as the initial treatment for adult GCT [9]. Platinum-based combination chemotherapy is also frequently administered [10]. As such, adult GCT has been associated with a median recurrence-free survival of only 5 years [11]. A substantial proportion of cases (9–48 %) develop recurrence, usually within the pelvis, within a median time of 4–7 years [1,12,13]. While distant spread is uncommon, several reports describe primary ovarian tumors presenting with significant pulmonary metastasis [14,15]; however, our case did not include evidence of prior abdominopelvic disease.

Ultimately, our patient closely resembles the first documented case of primary pulmonary adult GCT with respect to age and clinical history [6]. Unfortunately, both patients developed intrathoracic recurrence, and ours is currently receiving external beam radiation to the site of PET-avid disease (Fig. 1C) in addition to continued pembrolizumab and letrozole therapy. The propensity for locoregional recurrence is well-established for this tumor type [3], and the purpose for presenting this case is to draw comparisons with the first reported patient, such that we can now refer to multiple documented cases as evidence for heightened concern for local recurrence when treating this rare presentation of adult GCT.

## 4 Conclusion

Our case helps expand the anatomic distribution across which adult GCT is thought to arise. While the pathogenesis behind a primary pulmonary manifestation remains unclear, rare cases of this tumor originating within the thorax seem to share a clinical course that includes locoregional recurrence. As a result, treating physicians should consider careful surveillance for progressive disease when faced with this rare presentation of adult GCT.

## CRediT authorship contribution statement

**Mark G. Evans:** Writing – original draft, Methodology, Conceptualization. **Anthony Crymes:** Writing – original draft, Methodology. **Adam Bedeir:** Writing – review & editing. **David R. Braxton:** Writing – review & editing. **Matthew J. Oberley:** Writing – review & editing. **Robert T. Muldoon:** Writing – review & editing, Conceptualization.

## Disclosures

M.G.E. and M.J.O.: Caris Life Sciences (full-time employment, travel/speaking expenses, stock/stock options).

## Authorship

M.G.E. and R.T.M. performed study concept and design; M.G.E. and A.C. performed development of methodology and writing, review and revision of the paper; All authors read and approved the final paper.

## Funding

None.

## Declaration of competing interest

The authors declare the following financial interests/personal relationships which may be considered as potential competing interests: Mark G. Evans reports a relationship with Caris Life Sciences Inc that includes: employment, equity or stocks, speaking and lecture fees, and travel reimbursement. Matthew J. Oberley reports a relationship with Caris Life Sciences Inc that includes: employment, equity or stocks, speaking and lecture fees, and travel reimbursement. If there are other authors, they declare that they have no known competing financial interests or personal relationships that could have appeared to influence the work reported in this paper.
